# Historical Epistemology: a Research Approach in Psychiatry

**DOI:** 10.1007/s12124-020-09589-2

**Published:** 2020-12-04

**Authors:** Ivana S. Marková

**Affiliations:** Hull York Medical School, Allam Medical Building, University of Hull, HU6 7RX Hull, UK

**Keywords:** Historical epistemology, Descriptive psychopathology, Hybrid objects, Concepts

## Abstract

Drawing on a key issue raised in the paper by Scardigno and Mininni (2021), this commentary explores the question of historical research in psychiatry. Firstly, the importance of historical research is highlighted for both psychiatry as a medical discipline, and for descriptive psychopathology, the language of psychiatry. Of significance has been the construction of psychiatry as a hybrid discipline formed through the deep participation of both the natural and the social sciences. This in turn brings to light the fundamental difference in epistemological basis to psychiatry and medicine, with ensuing consequences for our understanding of mental disorders and for the development of further research methodology. Likewise conceptually hybrid, the special role carried by mental symptoms in psychiatry places them, as concepts, in the position of crucial research tools. Secondly, given some of the complexities raised in carrying out historical research in this area, the issue of how this should be approached is examined. The method proposed here is that of historical epistemology. This is an approach that focuses on concepts, on mapping their biographies in order to clarify their structures, their roles, their discontinuities, their relationships and interactions with other concepts and so on. Given the central role of concepts in psychiatry and descriptive psychopathology, this approach to their study is most likely to provide valid and meaningful results.

In their article ‘un-certainty as a pragmatic resource for psychiatric argumentation: a diachronical and diatextual approach’, Scardigno and Mininni (2021) present a historical analysis of 90 articles selected randomly from the British Journal of Psychiatry over the 160 years of the journal’s life. Using textual resources, they analyse how the way in which psychiatrists communicate their findings has changed over this time. Their diachronic approach is thus restricted to textual analysis and to predominantly Anglo-American psychiatry. Whilst, this may give rise to some interesting questions, its decontextualizing approach (e.g. from other medical writings, from social, cultural, political, economic, and other contexts) can only provide a very partial narrative. Amongst a number of issues raised by this paper, I therefore want to concentrate on two that are particularly pertinent to the study of psychiatry. Firstly, there is the question of why carry out historical research in this area; what is the purpose in doing this? Secondly, how should this be carried out, or what methods are most appropriate in order to yield epistemically valid and meaningful results?

## Why Do Historical Research?

The question I want to address here is not about why do historical research in general, but rather what is it about psychiatry, that makes historical research an important area of enquiry (Berrios 1994)? Before tackling this, it is important to make a distinction that is vital to addressing this question. This concerns the differentiation between psychiatry and descriptive psychopathology. Psychiatry is the discipline that deals with the understanding, assessment and management of patients with certain afflictions, currently termed mental disorders. Descriptive psychopathology on the other hand is the language of psychiatry, and refers to the terms and concepts that have been constructed to describe, capture and organise the mental and behavioural phenomena that constitute mental disorders (Berrios 1996). Psychiatry and descriptive psychopathology are thus quite different in structure and as such will demand correspondingly different approaches to their historical study and will yield different points of interest and importance from such studies. Here I briefly examine each from the perspective of this first question.

### Historical Research in Psychiatry

Psychiatry, or Alienism as it was originally called, has a relatively short history. It only became a medical speciality at the beginning of the 19th century. Before that, madness was a social category, conceived in a multitude of ways including as illness, as something evil or something divine, as a blessing, as supernatural etc. Consequently, madness was managed or dealt with in various ways and by a range of persons. Thus, people deemed to be mad might be locked up in prisons, asylums or hospitals or they might be ostracized/exiled or revered; they might be looked after by families, communities, physicians, the church, and so on (Porter 1997). That psychiatry came under the auspices of medicine was the result of complex socio-political processes in which the social institutions at the time participated to a greater or lesser degree. Differences in the socio-political processes across Europe meant that there were likewise major differences in the way that psychiatric systems developed in different European countries (Berrios 1996; Berrios and Porter 1995).

The medicalisation of madness has had major consequences for psychiatry, both for the way that psychiatry is organised as a discipline and the way it teaches and practises its subject, as well as, and crucially, for the way in which madness is understood and managed. Historical research in this area therefore becomes important for various reasons. Foremost, and perhaps most importantly, such historical research helps to clarify the epistemological basis to psychiatry as a discipline. In contrast to medicine, whose foundational basis as disorder of matter (the body) has been inherent to its definition for thousands of years, historical analysis reveals a very different foundation to psychiatry. Most obviously, it is evident that there is no such definitive origin or site to madness. Instead, history shows us that the decision as to whether behaviours and / or mental states are deemed abnormal is determined by society, by culture, and by the historical period in which the so-termed abnormalities are situated. Such determinations thus vary. What is considered abnormal in one culture may not be in another, or similarly behaviours viewed as abnormal in one historical time are viewed differently in another (Kirmayer et al. 2015). And, as testified by historical accounts, before the medicalisation of madness, interpretations of abnormality in peoples’ mental states and behaviours have been multifarious. What this indicates is that interpretation of human behaviours and mental states lies in a different epistemological plane to the interpretation of abnormality of the structural and physiological body. In other words, the epistemological basis to psychiatry is crucially different to that of medicine (Berrios and Marková 2015; Marková and Berrios 2012). An epistemology based largely on ‘meaning’ is different from that based on ‘matter’.

To deal with this, psychiatry had to develop as a hybrid discipline borrowing from both the natural sciences and the newly developing social sciences (Heilbron et al. 1998). The latter were emerging at that time as challengers to the natural sciences in seeking explanations for human beings and their place in the historical world (McDonald 1993). Thus, on the one hand, the medicalisation of madness meant that natural sciences were driving the understanding of madness as disease with a corresponding search for organic explanations of problems (Ackerknecht 1967). On the other hand, understanding abnormal behaviours, the meaning behind man’s actions, needed the help of social sciences, to make sense of these in the context of the individual’s history and circumstances. It was therefore only by developing as a hybrid discipline that psychiatry could incorporate such different approaches to making sense of various human behaviours.

In revealing this deeply hybrid structure of psychiatry, historical research marks an important epistemological turning point. This is because the hybridity carries vital consequences for our understanding of psychiatry and its objects of enquiry, namely, mental disorders and mental symptoms. In addition, however, it carries crucial implications for the way in which further research into psychiatry and its objects needs to be developed in order to obtain legitimate results. For example, it becomes apparent that research methods in psychiatry cannot be modelled entirely on research methods in medicine which are themselves modelled on the natural sciences (Marková and Berrios 2015, 2016).

Secondly, historical research is able to show the sorts of factors and processes involved in the construction of the discipline. Whilst this is not the place to explore these, what emerges from such work is that these factors are manifold and have little to do with changes in ‘scientific’ understanding. Instead what we see is that the driving forces for such change belong to the realm of politics, economics, fashions and other such areas in which sections of society are vying for different elements of control. In turn, understanding the influence of such processes draws attention to the temporality and instability of the disciplinary boundaries. Over the 200 years of its existence as a medical discipline, the boundaries of psychiatry continue to shift and stretch as socio-political factors exert their pull. Thus, whilst initially psychiatry dealt with madness (renamed psychosis after the 1860s), epilepsy and some organic disorders, over the course of the 19th century it expanded to include conditions later called the ‘neuroses’ such as anxieties, obsessive-compulsive disorders, panics, hypochondriasis, hysteria, and secondary depressions (López Piñero 1983). Similarly, in recent times we can see in the UK, for example, a growing permeability in the psychiatry boundary with the development and expansion in the past 10 years of the IAPT (Improving Access to Psychological Therapies) service. Here, the psychological discipline is taking over a large part of what previously was subsumed by the psychiatric discipline. Importantly, this change in disciplinary boundary has not been the result of new scientific understanding but a response to changes in societal needs and to economic factors (Layard et al. 2006).

Thirdly, beyond making sense of the transitory nature of psychiatry as a medical discipline and the processes involved in shaping its changes, historical research is necessary for understanding how the medicalisation of madness shaped the conception of madness and its management. When the mental states and behaviours deemed abnormal were brought under the umbrella of medicine, psychiatry adopted the clinico-anatomical model in order to understand, research and manage patients (Berrios 2008). This meant that from the beginning mental disorders or madness were conceived as diseases and psychiatrists sought to identify signs and symptoms, akin to those in medicine, which would help localise the associated pathology in the body. The essential point to note, however, is that this process of medicalisation does more than simply add another speciality to the discipline. Rather, it shapes a way of thinking and understanding the subject, creating in this case a conceptualisation that coheres with the wider medical outlook. In turn, subsumed within this framework, efforts at building further knowledge, the development of research directions and methodologies themselves become moulded and constrained by the new medical parameters. In other words, the whole thinking, the language and the practice around mental disorders become imbued with the medical paradigm. The language borrowed and taken over from medicine pushes and reinforces the same direction of thinking. For example, terms which had a specific meaning in the medical discipline become metaphorized in the psychiatric discipline, but ‘with continued use, once- novel metaphors become frozen, their original, metaphorical meanings become literal’ (Glucksberg 1999, p 536). In this way, psychopathology or pathology of the mind become as real and demanding of similar research exploration as pathology of the liver or heart. By unfolding this process, historical research can thus bring to light the origins of this disciplinary paradigm thereby uncovering the nature of the structures involved.

### Historical Research in Descriptive Psychopathology

Turning now to descriptive psychopathology as the language constructed to describe the mental symptoms and signs constituting mental disorders, the special importance of historical research in this area becomes apparent. As mentioned above, this language was needed when psychiatry became a medical discipline at the beginning of the 19th century. Hence its creation into a systematic organisation of symptoms and signs followed that of medical semiology (Berrios 1996, 2008). Prior to that, no such language existed. Historical exploration around the creation of this language however is necessarily different albeit overlapping with the historical exploration of the construction of psychiatry. This is because understanding the development of a language involves exploring factors and processes that are different from those involved in the development of a professional discipline. We saw earlier that psychiatry was constructed as a hybrid discipline, borrowing from both the natural and the social sciences in order to organise and manage the abnormalities of behaviours and mental states that made up the foundational core. Descriptive psychopathology thus likewise had to be conceptually hybrid. Symptoms became complexes of incongruent elements, constituted on the one hand by some form of material or organic change represented by some brain activity and on the other hand by ‘meaning’, a fluid-like, non-material element. Meaning in turn meant capturing and grouping portions of subjectivity which could then be organised into mental functions (Berrios 2008) with the help of concepts taken from the social sciences, linguistics, hermeneutics, etc.

The importance of doing such historical research however lies in the role that descriptive psychopathology carries in psychiatry. This is a role that is very different from the role that symptoms and signs carry in clinical medicine (Marková and Berrios 2016). The medicalisation of madness has bestowed upon it a common language thus giving the impression that analogous concepts are involved in both disciplines. This however is misleading since, as mentioned above, historical research shows that the epistemological basis to psychiatry and to medicine is radically different. This carries crucial implications for the nature of symptoms and signs. Importantly, the symptoms and signs in psychiatry are of a different kind to those in medicine. They are constitutive of mental disorders in a way that symptoms and signs in medicine are not. Thus, descriptive psychopathology carries an essential diagnostic role as well as an important therapeutic role (Marková and Berrios 2009, 2016). There are no biochemical or histological or radiological (or any other) markers that are diagnostic of mental disorders akin to those in medicine. Instead, making a ‘diagnosis’ or formulation of a mental disorder is entirely dependent on the elicitation of symptoms and signs, namely, the use of descriptive psychopathology in the relevant context of the person concerned. Similarly, as tools of communication, these symptoms and signs become incorporated in much of engagement work and therapeutic practices. The words, the language of symptoms and signs form the vehicle through which patients and clinicians make sense of particular experiences and are the medium through which changes can be effected.

Given this role for descriptive psychopathology in the determination, understanding and management of mental illness, historical research into the construction of this language is clearly vital. Its constitutive role means that it is only through understanding how this language developed, what factors have shaped and continue to shape its construction, that the structure of what we term mental disorder can be comprehended.

## How Should Historical Research Be Carried Out in Descriptive Psychopathology?

On account of the importance of carrying out historical research in both psychiatry and descriptive psychopathology, the question then is how this should be carried out. Even outlining some of the issues raised when considering the importance of historical research shows that this is an area beset by complexities. In the first place, we have to make sense of what sorts of factors might influence the development or the change to a particular object of enquiry. Such factors are multitudinous and heterogeneous spanning all areas affecting human life and meaning including e.g. societal, cultural, political, economic and environmental factors, and ranging from specific minor or major historical events to different personalities, etc. In the second place, changes across such diverse areas occur at different rates and hence influence change differentially, as depicted in Braudel’s long-, middle- and short-duration historical processes (Braudel 1970; Revel 2006). In the third place, interaction between such different areas as well as through time creates new factors and influences complicating interpretation. Finally, interpretation itself as to the significance of factors important in the influence of change is complex and affected by myriads of additional considerations. Historical exploration in a general sense thus involves a quagmire of issues that need to be teased apart. Where then to start with respect to psychiatry and descriptive psychopathology where already we have got a glimpse of the ever-changing panorama?

Focusing here on descriptive psychopathology since this lies at the core of our understanding of mental disorder, one approach that can be used to undertake a historical exploration in a meaningful and valid way is that of historical epistemology. Historical epistemology is a term that encompasses many different approaches to such exploration, but which share the view that scientific thinking and knowledge need to be understood in their historical development (Feest and Sturm 2011; Rheinberger 2010; Braunstein et al. 2019). These approaches thus tend to integrate, in different ways, methods from history of science, philosophy of science and history of philosophy. The historical epistemological approach taken in this instance is one that focuses on concepts as the objects of enquiry. As pointed out above, descriptive psychopathology is the language constructed to describe, elicit and organise changes in mental states and behaviours that are deemed to constitute mental disorders. As such, this language, amongst other things, is made up of concepts. Concepts are epistemological tools constructed to help organise pockets of reality. In this case, they are designed to deal with and capture ‘abnormalities’ in both subjective experiences and in objective behavioural expressions. The specific epistemological basis to psychiatry identified above, in which ‘meaning’ is the focal point, thus gives concepts a central role. In effect, concepts become the primary research tools here. Concepts in this sense have enormous power. Their constitutive role in psychiatry and psychopathology means that they are continuously guiding and shaping our understanding of mental symptoms and mental disorders. In addition, they not only dictate what happens to patients but they have a much wider remit. They determine future research directions and methodologies and therefore form the building blocks on which our knowledge in this area develops (Marková and Berrios 2016). Thus, it is these very concepts that need to be understood. The importance of this cannot be overstated. A way of understanding of a certain concept will carry wide ranging consequences for patient care, for clinical services and for the evolution of psychiatry as a discipline and its language, descriptive psychopathology.

A historical epistemological approach in this area is about exploring these concepts, about researching their biographies so that their roots can be examined, and the factors involved in putting them together can be identified and understood in their original and subsequent contexts. For various reasons, this is a complex research enterprise, not least because concepts themselves are heterogeneous, varying in all their aspects including their stability, longevity, complexity, their roles and functions, rates of change, the ways in which they cohere with other concepts and so on. This heterogeneity stems from the way that they are constructed. Examining this briefly here but elaborated elsewhere (Berrios 2011), concepts result from the historical convergences of (i) terms (names), (ii) theoretical accounts/explanations and (iii) the referents (objects of interest). All of these individual components, however, are subject to change as languages evolve, as explanations and theories are modified and as the ways in which referents are identified change in light of continually transforming contexts. Moreover, the changes often will affect the components independently, thus creating important discontinuities in concepts. For this reason, throughout history, we find that the same term has been used to name different concepts, or, different terms have been used to name the same concept. Similarly, referents have been indicated by different concepts.

### An Example

We can illustrate some of these issues by briefly considering the concept of trauma. Whilst there is no space here to bring out the details of a historical epistemological approach regarding trauma, some of the changes and discontinuities in the history of the concept can be highlighted. Thus, up until the end of the 18th century, the concept of trauma referred predominantly to lesion or wound. The referent was perceived as a break in anatomy and thus situated in time and space, was clearly visible, and carried stable ontology. However, during the course of the 19th century, in the context of changing views in medicine and disease and the growth of physiology as a separate discipline (Magner 2002)- subsequent to the construction of ‘function’ as an independent construct, then lesion started to refer not only to structural anatomy but also to physiology. This meant that the referent to trauma became more abstract as it no longer needed to be visible or static but was instead dynamic and was underpinned by different explanatory accounts. Then, at the beginning of the 20th century, in the wake of brain localization debates, the concept of psychological lesion developed, with ‘psychological’ disruption creating new underlying explanations and occurring at yet another level of abstraction. Subsequently, following his criticism of the brain localisation model for understanding psychological function, Freud changed the term ‘psychological lesion’ to ‘psychological trauma’ (Guenther 2013). The concept thus once more changed term and became underpinned by yet again very different sorts of explanatory narratives. Further changes continued in referent and explanatory accounts as later on, influenced by wider social, environmental and economic factors, the trauma concept became broader still (Micale and Lerner 2001). In these ways, shifts in one field of thinking influence changes in others such that the successive changes in terms, referents and explanatory accounts result in concepts that are very different from previous ones bearing the same name.

By mapping the biographies of concepts, historical epistemology is able to address the sorts of complexities raised above. It can identify conceptual continuities and discontinuities, their longevity, the ways in which they interact with each other, relate to and nest in other concepts, and explore the sorts of factors that govern such features. These factors which extend to social and political spheres can often only be partially understood as historical resources do not necessarily yield specific and/or explicit accounts of conceptual construction and change. It is however only by carrying out such research, that we can gain some understanding of the structures that constitute the language of psychiatry. Importantly however what such a research approach shows is that concepts are not invariant, that they have no unchangeable essences. Instead, built as they are to serve the needs of a society at a particular historical time, their meaning is understandable only in terms of that culture which constructed them.

## Conclusion

Whilst currently included as a medical speciality, its creation as a hybrid discipline places psychiatry in a different position from that of other clinical medical specialities. Constituted through the competitive forces of both the natural and social sciences, its language, descriptive psychopathology is likewise conceptually hybrid. Historical research in this area is important because, amongst other things, it brings to light this crucial finding, thereby exposing the fundamental difference between the epistemological basis underlying psychiatry and medicine. This in turn draws attention to concepts as core to our understanding of psychiatry as a discipline, central to its objects, mental symptoms and mental disorders, and as constituting the framework on which the language of psychiatry, descriptive psychopathology is based.

Research methodology that works well in the natural sciences and general medicine may not always be the most appropriate approach to take to a discipline whose objects of enquiry are deeply hybrid. Instead, seeking to explore such objects through their historical development and through the interplay of sociocultural, economic, political and other factors that continue to shape them is necessary in order to gain an understanding of their structures and the elements that constitute them. Meaning has to be elucidated first, before possible organic constituents and correlates can be validly sought. Historical epistemology is one such research approach that specifically addresses this.
